# Systematic Workflow Optimization for Ultra-sensitive Targeted Immunopeptidomics

**DOI:** 10.1016/j.mcpro.2026.101634

**Published:** 2026-08-04

**Authors:** Jonas D. Förster, Jonas P. Becker, Nika Vučković, Angelika B. Riemer

**Affiliations:** 1 Division of Immunotherapy and Immunoprevention, German Cancer Research Center (DKFZ), Heidelberg, Germany; 2 Molecular Vaccine Design, German Center for Infection Research (DZIF), Partner Site Heidelberg, Heidelberg, Germany; 3 Immunopeptidomics Unit, National Center for Tumor Diseases (NCT), NCT Heidelberg, A Partnership Between DKFZ and University Hospital Heidelberg, Heidelberg, Germany; 4 Faculty of Biosciences, Heidelberg University, Heidelberg, Germany

**Keywords:** immunopeptidomics, targeted mass spectrometry, parallel reaction monitoring (PRM), data-independent acquisition (DIA), human Papillomavirus (HPV), tumor antigens, method optimization

## Abstract

Epitope detection sensitivity remains a primary bottleneck in mass spectrometry (MS)-based immunopeptidomics, as conventional discovery-based workflows such as data-dependent (DDA) and data-independent acquisition (DIA) frequently lack the sensitivity required to detect ultra-low abundant targets. While these untargeted methods are powerful for mapping the general immunopeptidome, the stochastic nature of precursor selection and the presence of complex, chimeric spectra mean that rare species, such as viral or mutation-derived neoepitopes, often remain undetected. In this study, we present optiPRM+, an ultra-sensitive targeted-first workflow for the Orbitrap Exploris 480 platform that integrates systematically optimized targeted acquisition with untargeted DIA contextualization to bridge this sensitivity gap. Our approach centers on the empirical characterization of target peptides using direct infusion-MS to determine optimal fragmentation conditions and inclusion list-driven data-dependent acquisition (iDDA). To maximize signal-to-noise ratios for these trace-level targets, we employed ultra-high MS2 resolutions (up to 480,000), ion injection times up to 1000 ms, and narrow precursor isolation windows. Additionally, we discovered that precursors with a charge state exceeding their basic amino acid count require unusually low energies for optimal fragmentation, which is especially relevant for the non-tryptic peptides characteristic of the immunopeptidome. We applied the optiPRM+ workflow to the challenging biological case of Human Papillomavirus type 16 (HPV16), a virus known to suppress antigen presentation pathways. This optimized strategy enabled the confident identification and validation of the human leukocyte antigen (HLA)-A∗02:01-restricted epitope TIHDIILECV and, to our knowledge, the first MS-based detection of two novel viral targets: ISEYRHYCY (HLA-A∗01:01) and CVYCKQQLLR (HLA-A∗11:01). Subsequent global immunopeptidome analysis via DIA confirmed that these ultra-low abundance peptides were not detectable through untargeted methods despite being clearly validated by our targeted approach. By successfully detecting these viral peptides, we demonstrate that a systematically optimized targeted-first approach can uncover biologically relevant epitopes that remain invisible to conventional discovery-based workflows.

## Highlights

• optiPRM+ workflow pushes Orbitrap sensitivity limits for neoepitope detection.
• Ultra-high resolution and long injection times enable trace detections.
• Charge exceeding basic residue count requires lower collision energy.
• Targeted MS validates low-abundant viral epitopes missed by untargeted methods.

## Graphical Abstract

**Figure:**
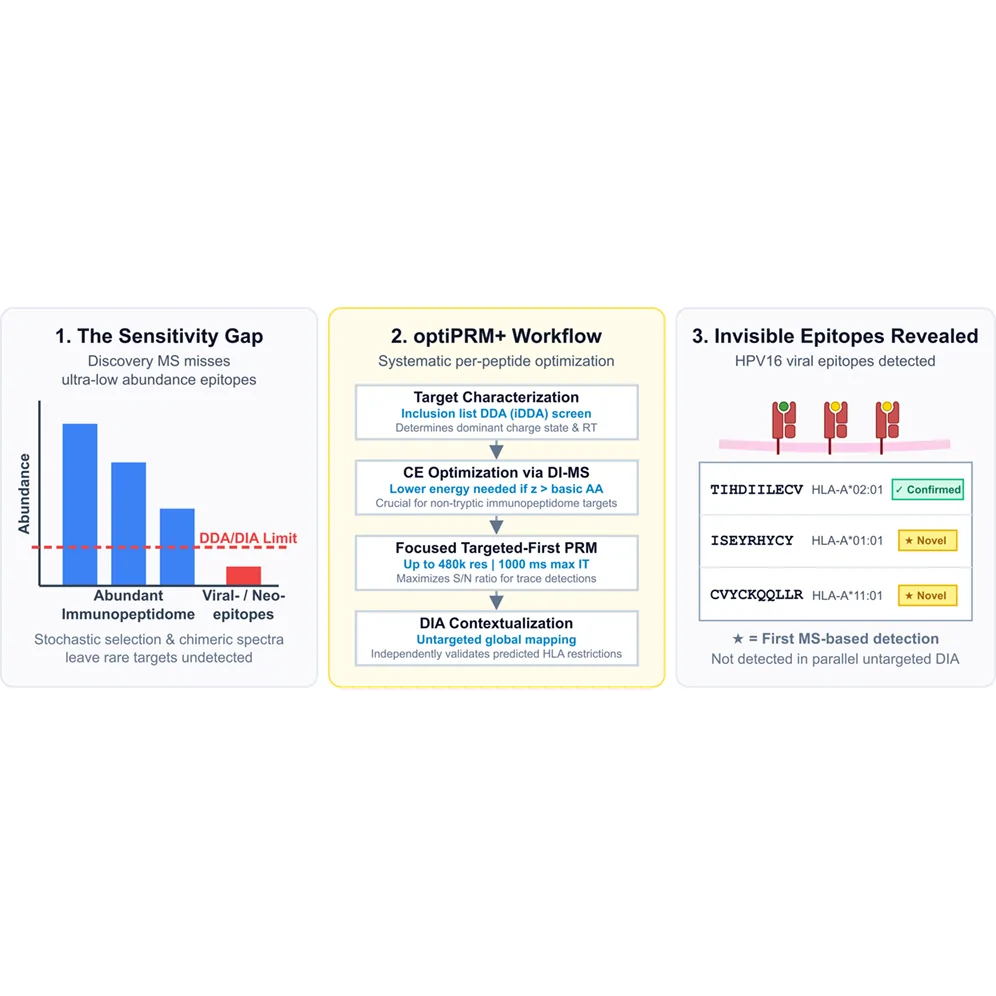

## In Brief

Low-abundance viral and tumor-derived epitopes often evade detection by conventional immunopeptidomics workflows, which lack sufficient sensitivity. We present optiPRM+, an optimized targeted mass spectrometry workflow, contextualized by DIA, that pushes Orbitrap sensitivity limits through specific parameter tuning, including a newly identified relationship between precursor charge state and optimal collision energy. Applied to HPV16, optiPRM+ validated a known viral epitope and achieved first-ever detection of two novel epitopes, demonstrating that a scalable targeted-first approach can uncover epitopes that conventional methods miss.

Epitope detection sensitivity is a recurrent and critical theme for mass spectrometry (MS)-based immunopeptidomics. While MS has become the gold standard for the direct and unbiased identification of peptides presented by HLA molecules (1, 2, 3), it currently lags behind the biological sensitivity of the immune system. Cytotoxic T lymphocytes (CTLs) have been reported to recognize as few as a single to a few hundred peptide-HLA complexes per cell (4, 5, 6), a level of sensitivity that may still effectively be 10^3^ to 10^6^ times higher than current generation MS instruments (7, 8). Consequently, while conventional discovery-based MS methods such as data-dependent (DDA) and data-independent acquisition (DIA) are powerful for mapping the general immunopeptidome, they often lack the sensitivity required for the reliable detection of ultra-low-abundant peptides. The stochastic nature of precursor selection in DDA and the complex, chimeric spectra in DIA mean that rare species, such as viral or mutation-derived tumor-specific neoepitopes, are frequently missed in favor of more abundant peptides (9, 10).

To address this sensitivity gap, targeted MS approaches, particularly parallel reaction monitoring (PRM), offer a powerful alternative. By focusing instrument acquisition time exclusively on a predefined list of peptide targets, PRM provides superior specificity and sensitivity (10, 11, 12). However, typical applications of PRM in immunopeptidomics are often limited to the validation of candidates identified by untargeted methods or to quantitative studies where maximizing raw detection sensitivity is not the primary objective (13). Consequently, workflows designed with the exclusive goal of detecting previously unobserved, ultra-low-abundant peptides are not widely described. We have postulated that systematic, peptide-specific optimization of instrument parameters can unlock significant gains in sensitivity that are not realized with standard, generalized settings. In our prior work, we introduced optiPRM, a workflow demonstrating that per-peptide optimization of collision energy dramatically improves detection sensitivity for target peptides, including mutation-derived neoepitopes (9).

In this study, we describe the scale-up of our systematically optimized targeted PRM-based immunopeptidomics workflow for maximal sensitivity on an Orbitrap Exploris 480 mass spectrometer (Thermo Fisher Scientific). We pair the targeted measurements with untargeted DIA analysis of the same cell lines for contextualization and independent validation of HLA restriction and refer to this integrated targeted-first workflow as optiPRM+, extending the per-peptide collision-energy-optimized optiPRM workflow (9).

To rigorously benchmark the sensitivity of such optimized workflows, we selected the oncoproteins E6 and E7 of Human Papillomavirus type 16 (HPV16) as a challenging biological test case. HPV16 is the most prevalent and oncogenic type of HPV, accounting for approximately 60% of cervical cancers and 85% of other HPV-driven cancers worldwide (14, 15). Its epitopes are exceptionally difficult to detect, making them an ideal model for "stress-testing" high-sensitivity MS methods. The virus has evolved numerous mechanisms to evade immune surveillance, primarily by maintaining viral protein expression at minimal levels and actively interfering with antigen processing and presentation pathways. These mechanisms include the downregulation of MHC-I surface expression and loading, the immunoproteasome, and the TAP transporter (16, 17). These immune evasion strategies profoundly alter the immunopeptidome and result in viral epitope abundances that represent a minute fraction of the total peptidome (16, 18). Consequently, state-of-the-art untargeted MS workflows frequently struggle to expand the repertoire of known presented E6 and E7 epitopes, with specific challenging targets consistently eluding detection (19). Furthermore, the viral oncoproteins E6 and E7—which drive malignant progression by inactivating tumor suppressors p53 and pRb (20)—are expressed at low levels and are of non-self origin, representing critical but elusive targets for epitope-centric immunotherapies (16).

We apply the optiPRM+ workflow to peptides selected from a previously established list of high-confidence HPV16 E6 and E7-derived HLA ligands (21), chosen as illustrative examples that span HLA alleles and peptide chemistries. By successfully detecting ultra-low-abundance viral peptides, we demonstrate that a systematically optimized targeted-first approach can bridge the gap between MS detection limits and the presentation levels of elusive T cell targets.

## Experimental procedures

### Cell Culture

The three HPV16-positive cell lines were chosen to maximize coverage of both HLA restrictions and peptide sequence motifs across the targeted set: CaSki and W12-20861 are widely used HPV16 models, while Goerke additionally contributes HLA-A∗01:01 coverage and non-tryptic peptide motifs. CaSki (RRID: CVCL_1100) cells were obtained from ATCC and were cultivated in Dulbecco’s modified Eagle’s medium (DMEM) supplemented with 10% fetal bovine serum, 1% penicillin/streptomycin and 2 mM L-glutamine. Goerke cells were obtained from Andreas Kaufmann (Charité) and were cultivated in Eagles’s MEM medium supplemented with 10% fetal bovine serum, 1% penicillin/streptomycin, 2 mM L-glutamine and 1% MEM NEAA. W12-20861 (22) cells were obtained from Paul Lambert (University of Wisconsin) and were cultivated in Ham‘s F-12 medium supplemented with 25% DMEM, 5% fetal bovine serum, 1% penicillin/streptomycin, 0.4 mg/ml hydrocortisone, 0.5 μg/ml cholera toxin and 5 μg/ml insulin. Cells were kept under standard conditions in a humidified incubator at 37 °C and 5% CO_2_. For the immunoprecipitation of HLA:peptide complexes, cells were washed with ice-cold PBS and harvested using Accutase (Thermo Fisher Scientific). Dry cell pellets were snap frozen in liquid nitrogen and stored at −20 °C until further usage. Cell lines were regularly authenticated and confirmed to be free of *Mycoplasma*, SMRV, or interspecies contamination by SNP profiling and multiplex-PCR by Multiplexion GmbH (23).

### Stable Isotope-Labeled (SIL) Peptide Mixtures

Crude synthetic SIL peptides were purchased from JPT Peptide Technologies (Berlin, Germany) or SynPeptide Co. Peptides were dissolved in DMSO to a concentration of 2 nmol/μl. Peptides were then combined into non-cysteine and cysteine-containing peptide mixtures of up to 50 peptides. These mixtures were designed to ensure that no precursors within charge states +1 to +3 would fall into the same m/z isolation window during direct infusion analysis. Aliquots containing 10 pmol of each peptide were prepared, dried by vacuum centrifugation, and stored at −80 °C until use.

### Immunoprecipitation of HLA:Peptide Complexes

HLA class I peptide complexes were isolated from cell pellets using a 96-well plate-based immunoprecipitation (IP) protocol based on previously published protocols (24, 25). Cell pellets were lysed in IP lysis buffer (0.25% sodium deoxycholate, 1% n-octyl-β-D-glucopyranoside (OGP), 1 mM PMSF, 1 mM EDTA, 0.2 mM iodoacetamide (IAA), 1x cOmplete Mini Protease Inhibitor Cocktail in PBS) for 1 h at 4 °C. Lysates were cleared by centrifugation at 40,000×*g* for 30 min at 4 °C. The IP was performed on a 96-well filter plate (Agilent) using a Waters Positive Pressure-96 Processor. Each well was loaded with 150 μl of W6/32 antibody-crosslinked Protein A Sepharose bead suspension. Cleared lysate (equivalent of up to 50 × 10^6^ cells per well) was loaded by gravity flow. Beads were subsequently washed 8 × with 1 ml IP wash buffer A (150 mM NaCl, 20 mM Tris-HCl, pH 8), 8 × 1 ml IP wash buffer B (400 mM NaCl, 20 mM Tris-HCl, pH 8), 8 × 1 ml IP wash buffer A, and 8 × 1 ml IP wash buffer C (20 mM Tris-HCl, pH 8). Peptides were eluted with 2 × 500 μl 0.3% trifluoroacetic acid (TFA). The eluate was reduced with 50 mM TCEP for 10 min, followed by alkylation with 400 mM IAA in the dark for 20 min. The peptides were desalted and purified using a Sep-Pak tC18 96-well plate (Waters). After sample loading, oxidation was performed by applying a freshly prepared 1:200 dilution of performic acid (5 μl of 30% H_2_O_2_ and 45 μl of 10% formic acid). Peptides were eluted with 28% acetonitrile (ACN)/0.1% TFA, dried in a vacuum concentrator, and stored at −20 °C.

### LC-MS Setup

All analyses were performed on an UltiMate 3000 HPLC system coupled to an Orbitrap Exploris 480 mass spectrometer (Thermo Fisher Scientific) via a Nanospray Flex ion source. Peptides were separated on a nanoEase M/Z Peptide BEH C18 column (130 Å, 1.7 μm, 75 μm × 200 mm, Waters) at a flow rate of 300 nl/min using gradients from 1% to 80% solvent B (ACN, 0.1% FA) over up to 110 min. Dried peptide samples were resolubilized in 5% ACN/0.1% TFA. For dynamic retention time (RT) alignment, samples were spiked with 50 fmol of Pierce Peptide Retention Time Calibration Mixture (PRTC, Thermo Fisher Scientific) and 5 fmol of a commercial retention time standardization kit (PROCAL, JPT Peptide Technologies, (26)) per injection. Electrospray ionization was driven with an applied potential of 2500 V. A pulled emitter (CoAnn Technologies) with 20 μm inner diameter (ID) to 10 μm ID at the tip was used.

### Direct Infusion MS

Collision energy optimization was performed according to our previously published optiPRM workflow (9). Dried synthetic peptide mixtures (10 pmol per peptide) were resolubilized in 5 μl of 50% ACN/4% FA. The sample was introduced into the mass spectrometer via a fused silica capillary connected to a syringe, with flow initiated by applying consistent pressure. A stable spray was achieved with an applied potential of 2500 V. For each precursor (charge states +1 to +4), MS2 spectra were acquired at 45k resolution across a normalized collision energy (NCE) range of 4% to 42% in 2% steps, with five iterations per NCE value in a randomized order.

### LC-MS Acquisition

Across all runs, the RF Lens was set to 50%, Automatic Gain Control (AGC) was enabled, lock mass was the polydimethylcyclosiloxane ion ([(Si(CH_3_)_2_O)_6_+H]^+^,445.120025 m/z) from ambient air in timed mode for the first 3 min.

For initial characterization of synthetic SIL peptides, an inclusion list-driven data-dependent acquisition (iDDA) method was used. A full MS1 scan was acquired at 120,000 resolution at 200 *m/z* over a mass range of 300 to 1650 m/z, with a 3e6 AGC target and 35 ms maximum injection time (max. IT). Precursors from the inclusion list were selected for MS2 fragmentation (custom NCE values determined by direct infusion, 15,000 resolution, 2e5 AGC target, 120 ms max. IT) to determine their RT and dominant charge states.

For targeted validation of endogenous peptides, a parallel reaction monitoring (PRM) method was employed. The full MS1 scan was set to 60,000 resolution (220–1450 m/z, 3e6 AGC, 25 ms max. IT). MS2 data were acquired in scheduled windows (up to 3.5 min around the experimentally determined RT) using custom NCE values determined by direct infusion. Precursor isolation was set to the *narrow* minimum vendor recommendation: 0.4 m/z for precursor ≤400 m/z, 0.7 m/z for ≤700 m/z, 1 m/z for ≤1000 m/z, 1.5 m/z for ≤1500 m/z, 2.0 m/z for ≤2000 m/z, 3 m/z for ≤2500 m/z. To maximize sensitivity, MS2 scans were acquired at 60,000 to 480,000 resolution with a 1e6 AGC target and a dynamic max. IT calculated to ensure 5 to 7 points across the chromatographic peak. The instrument's dynamic RT feature, indexed to the spiked-in PRTC peptides, was active to adjust scheduled windows during the run. Negative control runs consisting of a BSA digest were performed before sample analysis to monitor for LC carryover, and negative control peptides derived from mouse proteins were included in the target list for HLA-A∗02 assays to assess specificity.

For global immunopeptidome analysis, a data-independent acquisition (DIA) method was used. The method consisted of one full MS1 scan (120,000 resolution, 300–1650 m/z, 3e6 AGC, 35 ms max. IT). This was followed by 44 consecutive MS2 scans using dynamic precursor isolation windows covering the 299.5 to 1650.5 m/z range with 1 m/z overlaps. MS2 spectra were acquired at 30,000 resolution with 30% NCE, a 3e6 AGC target, and an automatic max. IT. The 44 variable precursor isolation-window widths are provided in Supplementary Table S2, and the method cycle time was set to “Auto”.

To assess the impact of sample load on chromatography, a HeLa protein digest standard (Pierce HeLa Protein Digest Standard, Thermo Fisher Scientific) was injected at increasing amounts (50 ng to 800 ng) and analyzed in DIA mode to monitor peptide RT shifts.

To test the effect of MS2 parameters on detection sensitivity, 20 ng HeLa protein digest standard mixed with up to 0.17 fmol/μl (1:30) of the PROCAL peptides were injected. PRM was used with the full MS1 scan set to 120,000 resolution, 360 to 803 *m/z*, 3e6 AGC target, 250 ms max. IT. The dynamic RT feature was off. 24 peptides of the 40 peptides in the PROCAL peptide mix were targeted, with the RT schedule defined such that none of the acquisition windows overlapped. Base settings for target peptide MS2 were scheduled acquisition with 30% NCE, 120,000 resolution, automatic scan range, 1e6 AGC target, 25 ms max. IT, scheduled target cycling for 2.1 s. Only parameters to be tuned were adjusted where needed.

### Data Analysis

Targeted PRM data were analyzed using Skyline (v. 24.1) (27). Following the fit-for-purpose framework for targeted MS assays (28), these targeted analyses correspond to Tier 3: although stable isotope-labeled (SIL) reference peptides were used, they served for identity confirmation rather than quantification, were acquired assay-externally rather than spiked into every run, and the workflow is exploratory and sample-specific. The precursor ions and their monitored transitions for each analyte are listed in Supplementary Table S1. MS2 chromatograms were extracted with a 3 to 7 ppm mass tolerance. For a signal to be considered a confident detection, peaks were required to have a normalized spectral contrast angle (NSA) (29) score ≥0.85 based on at least five specific fragment ions (≥3 amino acids, types y, b, a) when compared to a spectral library generated from SIL reference peptide acquisitions. Data were exported as csv tables for downstream analysis and visualization in R (v. 4.3.3) using the tidyverse suite of packages (v. 2.0.0). The acquisition parameter test used 3 ppm mass tolerance for all but the resolution test, which used 7 ppm to accommodate the lower precision at a resolution of 60,000 at 200 m/z. For producing the transition count, the Skyline document was adapted to include all ions (≥1 amino acids; types y, b, a) that were detectable in the reference library. Analysis was performed in R. Untargeted DIA data were analyzed using Spectronaut (v. 17.6, Biognosys) with directDIA (library-free) and the UniProtKB/Swiss-Prot human reference proteome (retrieved 2021-10-21; 20,387 entries) combined with the HPV16 reference proteome (retrieved 2021-10-21; nine entries). Settings were automatic tolerances, unspecific digest mode and a maximum of 2 PTMs per peptide. Carbamidomethyl (C) was set as a fixed modification. Acetylation (N-term) was set as variable modification. Oxidation (M) was by default set as fixed modification. Identifications were filtered to 1% PSM and 1% peptide FDR. For all detections, binding predictions for the respective HLA class I alleles of the sample were generated with netMHCpan (v. 4.1b, (30)). Data from direct infusion experiments were analyzed using custom R scripts to determine the optimal NCE that maximized the signal of the fourth-sixth most intense fragment ions, ensuring at least six transitions were detectable. The R scripts are published on GitHub at https://github.com/jonasfoe/ms_targeted_workflows.

### Experimental Design and Statistical Rationale

For each cell line, cells were expanded in culture and harvested as separate cell pellets that were processed in four independent immunoprecipitation (IP) experiments, yielding four technical replicates per cell line. Three of these replicate eluates were analyzed by targeted PRM to assess the reproducibility of each peptide detection, and the fourth was analyzed by untargeted DIA to contextualize the targeted detections within the global immunopeptidome. This design was identical across all 3 cell lines (CaSki, Goerke, and W12-20861). Because the replicates derived from overlapping culture expansions rather than from independently cultured biological samples, they constitute technical rather than biological replicates; accordingly, the statistical rationale of the study is the reproducible detection of low-abundance target peptides across independent IP and acquisition replicates. Confident detection was defined by a normalized spectral contrast angle (NSA) ≥ 0.85 against the reference spectrum (see Data Analysis), and each reported epitope was further confirmed by a synthetic SIL spike-in.

## Results

### Target Peptide Characterization

**Fig. 1:**
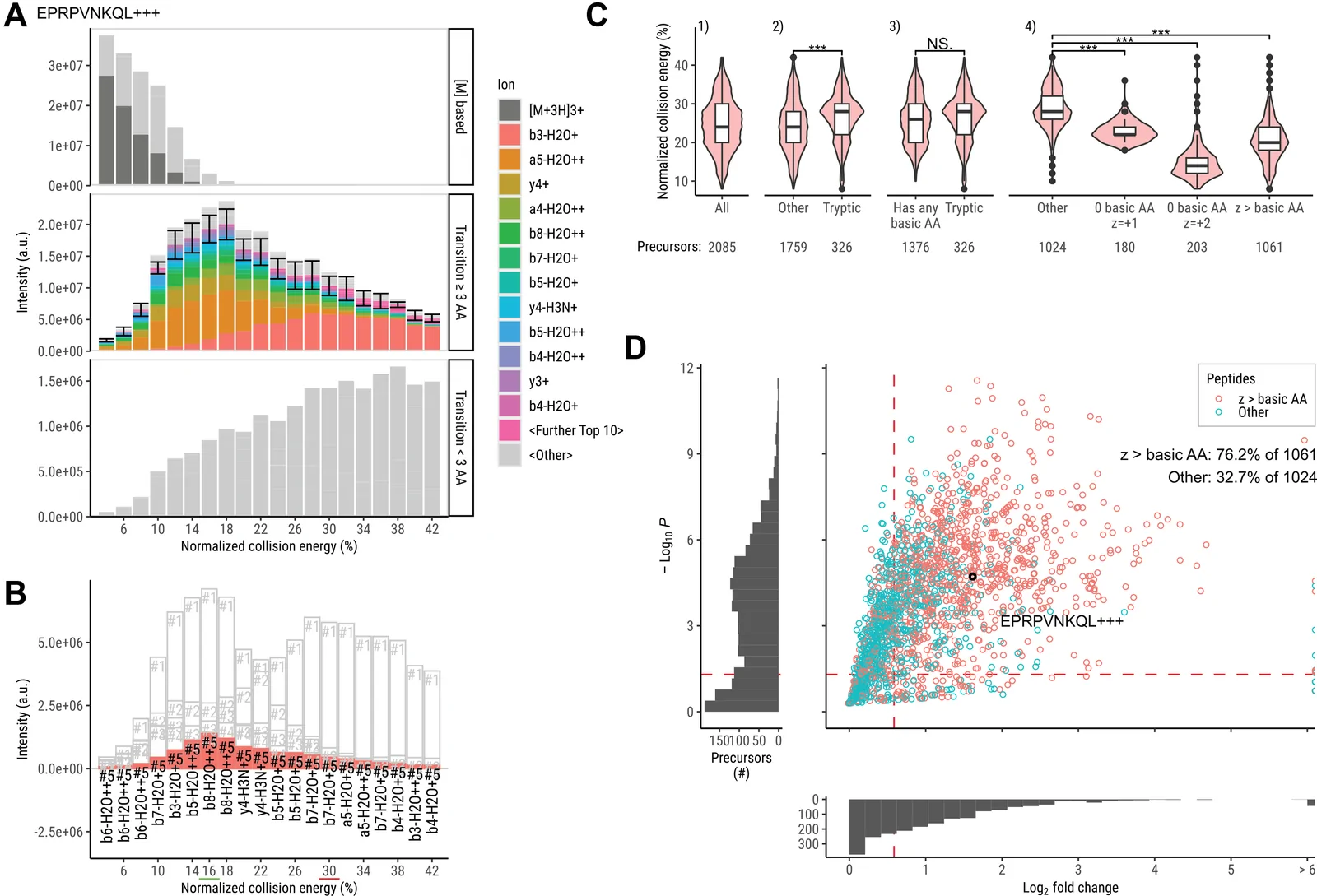
**Collision energy optimization for target peptides.***A*, Stacked quantification of MS2 signal acquired via DI-MS for peptide EPR[+10]PVNKQL[+7] at precursor charge state +3 over a range of normalized collision energies (NCEs). For each NCE, five spectra were acquired and mean ion intensities are shown. The main focus is on highly specific fragments of length ≥3 AA and the most dominant fragments are colored. Ions of lower specificity are separated as precursor ([M]-based) and of length <3 AA. Confidence intervals are the standard deviation of the sum of the *top* 10 ions. (*B*) Overlaid (not stacked) mean intensity of the top five ions from (*A*) at each NCE. The rank #5 ion is highlighted in *red* and was used to pick the optimum. The default NCE of 30% is marked in *red* and the optimized value for the precursor of 16% is marked in *green*. *C*, the optimized collision energies as determined for 2085 precursors split according to different factors relating to basic amino acid content of the peptides and the precursor charge z. Confidence is indicated according to an unpaired two-sided Welch’s *t* test. *D*, optimization results for all relevant precursors. Compared is the signal at a standard NCE of 30% to the optimal value for each precursor at rank #5 as in (*B*). Histograms for the x- and y-axis show the overall distribution of points (precursors) in the plot. Precursors in the *top-right* corner, delimited with *dashedredlines*, show a signal gain ≥50% at a *p*-value <0.05 according to an unpaired one-sided Welch’s *t* test with 4.5 degrees of freedom. The location of the peptide analyzed in (*A*) is indicated. The percentage of precursors appearing in the *top-right* section and an overall count are indicated to the *right* of the panel.

To perform a parallel reaction monitoring (PRM) assay that is fully optimized for sensitivity, we aimed to first characterize each target peptide by analyzing its synthetic stable isotope-labeled (SIL) variant. The initial step, which can be performed before any LC-MS characterization, is to employ our optiPRM pipeline for collision energy optimization (9). This direct infusion MS (DI-MS) method is performed in a scalable fashion with peptide mixtures and ensures that optimal collision energies are used for our putative HLA-presented target peptides, which are often non-tryptic and thus tend to fragment differently from canonical tryptic peptides. MS2 spectra are acquired across a range of NCE values and analyzed with a focus on maximizing fragments of at least three amino acids in length to ensure specificity (Fig. 1*A*). We defined the optimal NCE as the value that maximized the signal for a moderately intense fragment ion (e.g., the rank #5 ion, Fig. 1*B*), thereby promoting a balanced fragment ion spectrum suitable for both confident identification and robust quantification via extracted ion chromatograms (XICs). In this study, we refined our previous dataset (9) to include optima for 2085 precursors. This larger dataset confirmed our earlier findings that non-tryptic peptides benefit more from CE optimization than tryptic ones (Fig. 1*C*, panels 1–2). While tryptic peptides, with their C-terminal basic residues (K/R), readily generate informative y-ion series upon HCD fragmentation, we found that the presence of a basic residue at any position did not significantly alter the CE optimum (Fig. 1*C*, panel 3). Instead, a more detailed analysis revealed that the most significant factor driving CE optima to lower energies is when a precursor’s charge state (z) exceeds its total count of basic amino acids (R, K, H) (Fig. 1*C*, panel 4). Unexpectedly, we observed that these "overcharged" states were often the most intense species for many peptides under our standard LC-MS conditions. For example, the precursor for the peptide shown in Figure 1*A* has a dominant +3 charge state, which satisfies the z > basic AA count criterion and shows a fragmentation optimum at a low NCE; in contrast, the less intense +2 charge state for the same peptide has a more conventional optimum around 26% NCE (Supplementary Fig. S2*E*). Across our entire optimization dataset, 76.2% of precursors with z > basic AA count showed a significant benefit from CE optimization, compared to only 32.7% of other precursors (Fig. 1*D*). This effect is particularly relevant for immunopeptidomics, where peptides are not guaranteed to possess a C-terminal basic residue.

**Fig. 2:**
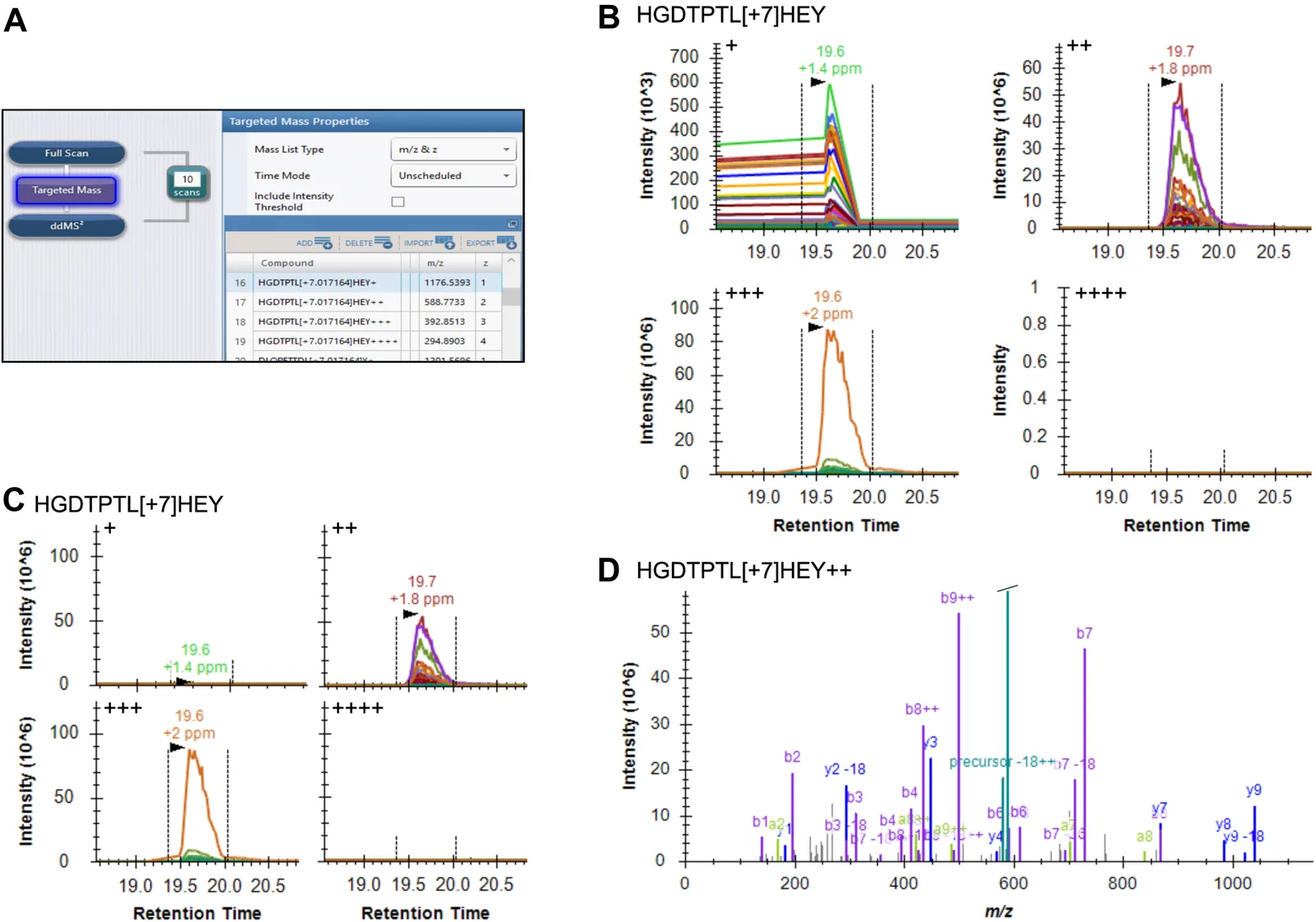
**Initial peptide acquisition through inclusion list DDA.***A*, method scheme in Xcalibur method editor for Orbitrap Exploris *B*, MS2 XICs for synthetic peptide HGDTPTL[+7]HEY for four charge states (+–++++) as produced by iDDA. Artefactual stretched lines are the result of processing with Skyline. *C*, XICs from *B* with unified y-axis for comparison of intensities. *D*, exemplary spectrum from z = +2 XIC in (*B*) at 19.7 min as annotated by Skyline. Precursor signal in center (turquoise) is pruned.

Next, employing LC-MS allowed us to acquire accurate retention time (RT) and precursor charge state characteristics for our target peptides. To this end, an inclusion list data-dependent acquisition (iDDA) workflow targeting the synthetic stable isotope-labeled (SIL) variants was used (Fig. 2*A*). This approach, natively supported by the instrument control software, performs triggered MS2 scans only for a predefined list of precursors, enabling their acquisition with higher sensitivity and temporal resolution compared to conventional DDA. The resulting data allowed for the high-quality reference XIC, including confident determination of indexed empirical RTs (Fig. 2*B*) and dominant precursor charge states (Fig. 2*C*) for our target peptides. This detailed characterization also provided an in-depth understanding of peptide elution behavior, which in some cases diverged from the standard model of a single chromatographic peak per peptide. For instance, some peptides presented multiple co-eluting charge states with competitive intensities. Furthermore, specific chemical properties led to peak splitting; a methionine-containing peptide produced two distinct peaks, likely due to the presence of diastereomeric sulfoxide forms (Supplementary Fig. S1*A*), while a proline-containing peptide exhibited cis-trans isomerization in its synthetic form, also resulting in two peaks (Supplementary Fig. S1*B*). Interestingly, the natural (endogenous) proline-containing peptide was detected as a single species, suggesting it exists in a single stable conformation *in cellulo*. Based on these observations, we adopted the strategy of targeting all peaks of significant intensity observed for a given synthetic peptide. The ability to visualize the high-resolution MS2 spectra acquired via iDDA directly within the Skyline software interface further assured confident identification of the synthetic peptides (Fig. 2*D*). While Skyline also offers *in silico* spectra prediction, we found it is generally not needed when using iDDA with purely synthetic samples. From these data, Skyline supports the creation of spectral libraries that can be used for PRM analysis. Alternatively, an additional heavy-peptide PRM run utilizing optimized CEs and retention times determined by the iDDA measurement and processed in the same way with Skyline can provide an optimally resolved reference signal.

### Optimization of LC-MS Acquisition

**Fig. 3:**
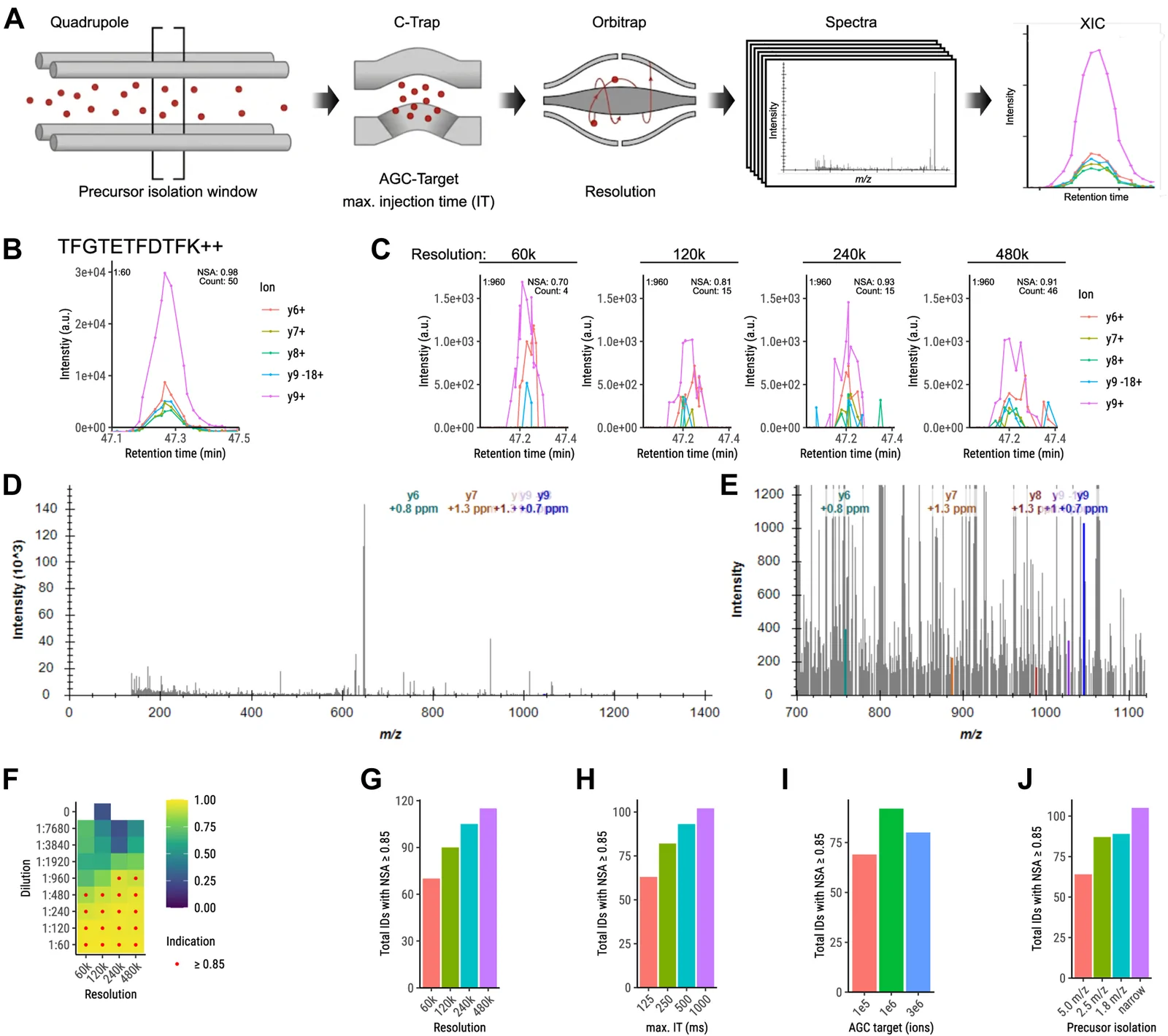
**MS scan parameter tuning.***A*, ion detection performance for ion chromatogram extraction in orbitrap PRM is determined by the interplay of four method parameters, affecting quadrupole isolation, ion collection in the C-trap, and Orbitrap scan resolution. (*B*) Reference XIC for synthetic peptide TFGTETFDTFK at z = +2, acquired at a 1:60 dilution against a complex matrix of tryptic HeLa digest at 480,000 resolution, representing a high-quality detection (NSA 0.98). Annotation as in (*C*). *C*, example for effect of scan resolution on the acquired XIC for synthetic peptide TFGTETFDTFK at z = +2. 1:960 diluted against a complex matrix of tryptic HeLa digest. Annotated at the *top left* of each panel is the dilution. Annotated at the *top right* are the NSA and the total amount of distinct peptide-specific ions detected. *D*, exemplary spectrum from rightmost XIC in (*C*, 480k) at 47.20 min, using a precursor isolation window of 2 m/z. Highlighted are the *top* five fragment ions. (*E*) Same spectrum as (*D*) but zoomed on specific ions. *F*, full panel of NSA results for the precursor in (*C*), with increasing difficulty of detection due to increased dilution from *bottom* to *top*. *G*, sum of total successful detections (NSA ≥0.85) for all valid peptides at all dilutions for the different resolutions tested. *H*, evaluation of injection time parameter as in (*G*). (*I*) Evaluation of AGC target parameter as in (*G*). *J*, evaluation of the precursor isolation parameter as in (*G*).

To further maximize sensitivity for detecting low-abundance peptides, we systematically optimized four key Orbitrap PRM acquisition parameters that govern MS2 ion detection performance: the quadrupole precursor isolation window, the interdependent maximum injection time (max. IT) and automatic gain control (AGC) target, and the Orbitrap MS2 resolution (Fig. 3*A*). In conventional PRM these parameters are held near balanced defaults mainly to preserve cycle time when many peptides are multiplexed, since adequate sampling of each elution profile bounds how long a single scan may take (31, 32, 33); this couples per-scan sensitivity (injection time, resolution) to throughput. When only a few peptides are targeted, cycle time is no longer constraining and this coupling breaks, allowing injection time and resolution to be driven to their instrument maxima. Far from costing sensitivity, this increases it, since S/N scales with the square root of acquisition time (34). The AGC target is the exception, bounded by a physical space-charge ceiling rather than by throughput. Using a standardized assay consisting of a peptide kit of synthetic peptides serially diluted against a constant complex matrix of a tryptic HeLa digest, we evaluated performance by monitoring the normalized spectral contrast angle (NSA) and the number of detected transitions. To allow for assessment of the maximal detection performance of the instrument, we selected 24 of the 40 peptides in the kit such that every one of them could be targeted exclusively for its entire elution time. Our investigation of MS2 resolution revealed clear sensitivity gains extending well beyond the 60,000 (at 200 m/z) setting typically sufficient for ion discrimination. For example, at a 1:960 dilution, an assay at 60,000 resolution detected only three of the top five fragment ions, prohibiting confident identification. In contrast, resolutions of 120,000 and above detected all top five ions with an improved NSA, and at 480,000 resolution, an unexpectedly high number of 46 distinct transitions were observed across the peak (Fig. 3*C*, transitions not shown, count readout from skyline). Detection here is based on the extracted ion chromatograms (XICs) of the targeted transitions rather than on the full MS2 spectrum; for reference, the same peptide at a 1:60 dilution and 480,000 resolution produces a fully formed, high-quality XIC with the expected fragment-ion hierarchy (NSA 0.98; Fig. 3*B*). This high transition count was not used for quantitation. Most additional fragments lie near the noise level or are elevated by off-target signal, so the NSA was restricted to the top fragment ions, and the dose-dependence of the total count is reported only as a supplementary observation. The per-fragment extracted-ion intensities underlying the NSA calculations for this peptide are provided in Supplementary Table S3. Interestingly, at this limit of detection, the underlying full MS2 spectra became highly uninformative and were dominated by off-target signals (Fig. 3*D*). However, zooming in on the specific m/z values for the targeted transitions revealed the clear, specific ion signals that constituted the high-quality XIC peak (Fig. 3*E*). Evaluating the full dilution series for this peptide confirmed that our NSA threshold for confident detection was reached at lower concentrations (i.e., with higher sensitivity) when using higher resolutions (Fig. 3*F*). This trend held true across the entire peptide set, where maximizing the resolution to 480,000 yielded the highest number of total positive identifications across all dilutions and peptides (Fig. 3*G*, Supplementary Fig. S3). Similarly, probing injection times revealed a clear benefit from using the longest possible max. IT of 1000 ms (Fig. 3*H*, Supplementary Fig. S4). The effect of the AGC target was non-monotonic: performance peaked at 1e6 and declined at the highest target (3e6), consistent with the overfilling and space-charge behavior expected at high ion populations (31, 34) (Fig 3*I*, Supplementary Fig. S5). We therefore used 1e6 for sensitivity-optimized assays. Finally, we found that using the narrowest manufacturer-recommended precursor isolation window for a given precursor mass consistently improved detection metrics by increasing specificity and reducing interference. Narrowing the isolation window is expected to reduce ion transmission, which is why a wider default is conventionally chosen to balance selectivity against transmission (33). In our hands, however, this specificity gain outweighed any transmission loss across the full dilution series (Fig 3*J*, Supplementary Fig. S6). Based on these results, we defined a set of "peak sensitivity" settings (480,000 resolution, 1000 ms max. IT, 1e6 AGC target, and narrow isolation window) to be employed for assays targeting a small number of peptides, while recognizing that these parameters can be and often were dialed back to reduce cycle times when a larger number of peptides are targeted simultaneously.

**Fig. 4:**
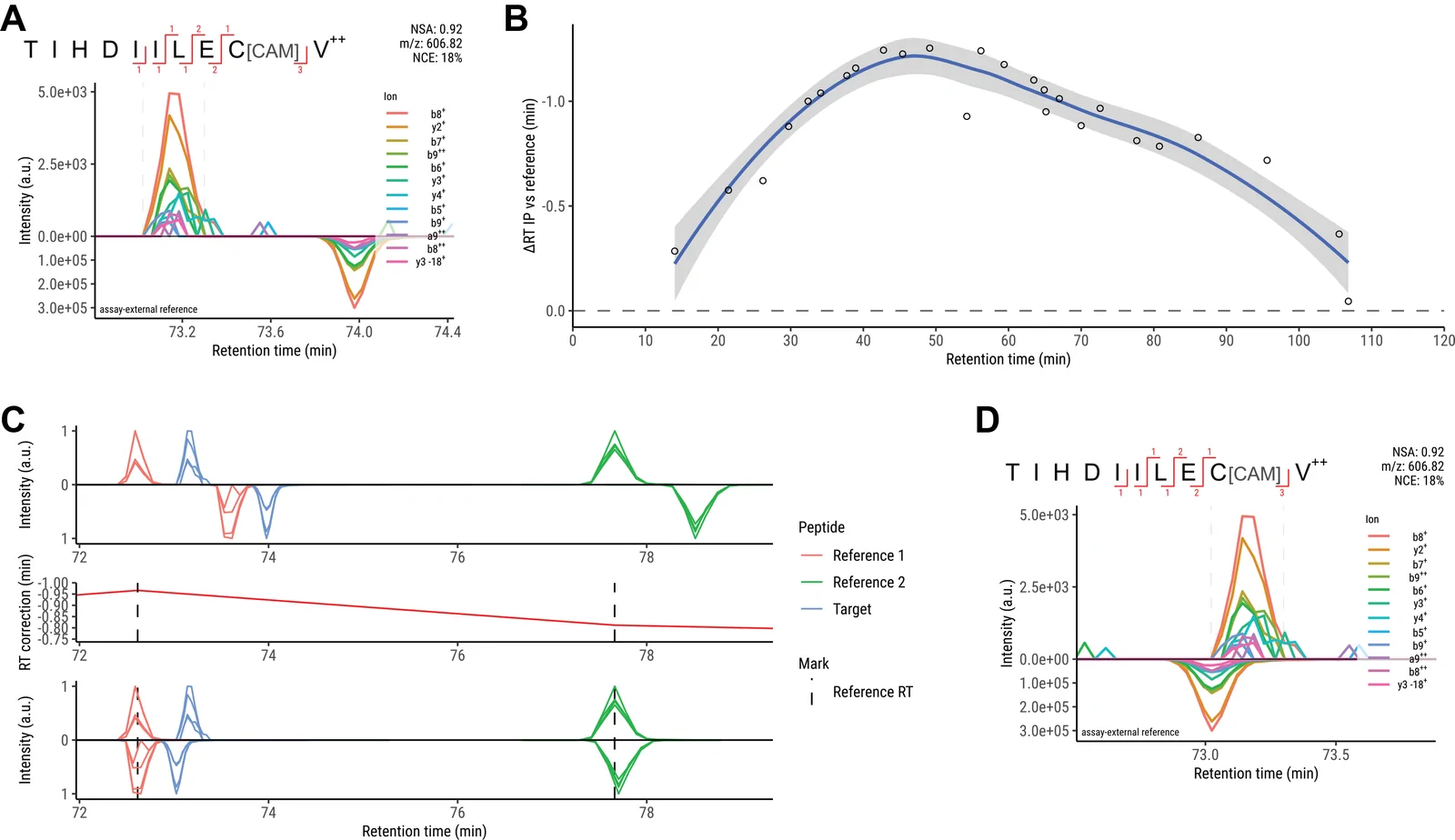
**HLA IP retention time shifts and alignment using standard peptides.***A*, MS2 XIC produced from an HLA IP with mirrored reference XIC. The reference XIC is shown with RT as-is. *B*, retention time shift of spiked-in reference peptides observed with an HLA IP, compared to an LC control acquisition immediately before. The dashed reference line at delta 0 reflects expected elution according to the LC control acquisition. Negative delta indicates early elution in HLA IP. The x-axis is that of the HLA IP. The method for the blue trendline is locally weighted scatterplot smoothing (Loess) in R with formula y ∼ x. *C*, scheme for RT correction of (*A*) using linear interpolation between adjacent reference peptides (*red*, *green*). Peak maximum intensities are normalized to 1. Above the x-axis are peaks from the HLA IP. Below x-axis are peaks from the reference acquisition. *Top panel* is the unaligned case. *Bottom panel* is the realigned case. *D*, MS2 XIC from (*A*) with realigned mirrored reference XIC.

The challenge of managing and titrating hundreds of synthetic reference peptides for internal standardization, along with the risk of light-form contamination from stable isotope-labeled (SIL) standards (35, 36), led us to perform initial discovery assays without assay-internal spike-ins. This strategy necessitated a robust method for managing inter-run retention time (RT) shifts. We frequently observed significant RT discrepancies between the endogenous signal in a complex peptide eluate after HLA immunoprecipitation (IP) and its corresponding assay-external synthetic reference, even when the runs were performed on the same day, precluding explanations like column aging (Fig. 4*A*). By comparing the RTs of standard peptides spiked into both blank runs and subsequent high-load immunopeptidomics runs, we observed systematic and load-dependent early elution of peptides (negative ΔRT) in the presence of a complex matrix (Fig. 4*B*). Further investigation using serial injections of tryptic HeLa digest at increasing loads confirmed that high peptide load on the column induces a transient, early elution of analytes (Supplementary Fig. S7*A*) without drastically altering peak widths (Supplementary Fig. S7*B*). Given these observations, we developed a robust, two-pronged strategy to manage RT variability without the necessity of modifying the LC system. First, we employed the instrument's built-in dynamic RT feature during acquisition, and second, for data visualization, we applied a post-acquisition RT correction to the external reference chromatograms based on linear interpolation between adjacent RT standard peptides (Fig. 4*C*). This alignment strategy proved effective, enabling good alignment and allowing us to place high confidence in trace-level signals despite considerable RT shifts between runs (Fig. 4*D*).

### Detection of Viral Epitope Presentation on Cell Lines

**Fig. 5:**
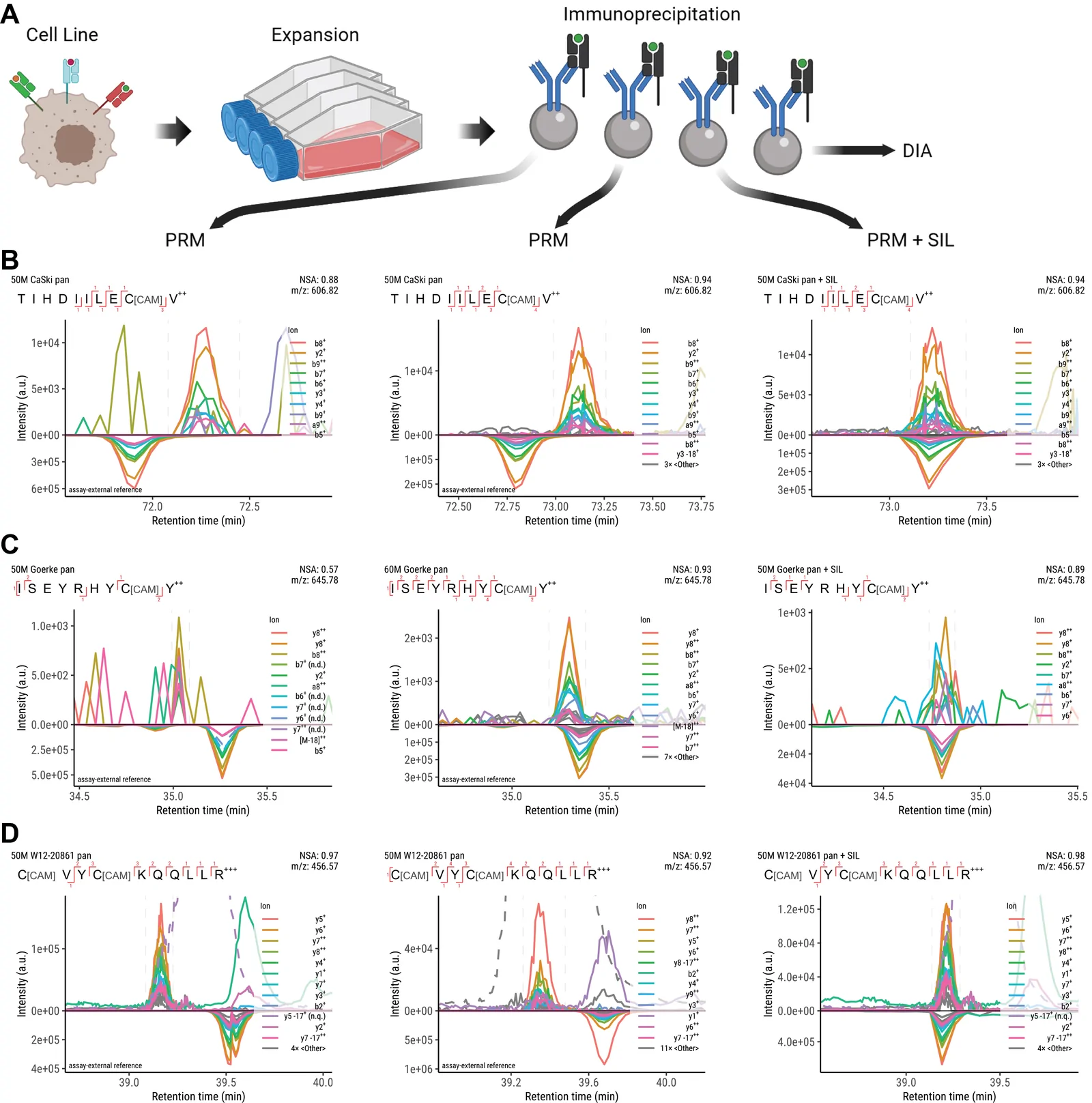
**Targeted detection of novel HPV immunopeptides.***A*, schematic of the experimental design for each cell line: cells are expanded in culture and processed in four independent immunoprecipitations (IPs), of which three are analyzed by targeted PRM (one with a synthetic SIL spike-in) and one by untargeted DIA. In panels (*B*–*D*), the colored XICs are the MS2 extracted ion chromatograms. The extracted ions, including further ions <Other>, are the known fragmentation products according to the synthetic reference acquisition. The assay-external reference signal is mirrored below for comparison. The NSA quantifies the similarity of the fragmentation pattern to the reference. *B*, progression from initial detection (*left*) and re-detection (*center*), which display an assay-external reference XIC for comparison, to absolute RT confirmation using synthetic SIL peptide spike-in (*right*) for peptide TIHDIILECV. *C*, similar progression as in (*B*) for peptide ISEYRHYCY. *D*, similar progression as in (*B*) for peptide CVYCKQQLLR.

Applying our fully optimized targeted workflow, we confirmed the HLA class I presentation of HPV16 E6/E7-derived viral epitopes. These peptides were drawn from a previously established, *in vitro* binding-validated ligand set (20): 157 distinct candidate peptides were targeted across the 3 cell lines, restricted to match each line's HLA alleles. Because the used ligand set reflects *in vitro* binding rather than confirmed cell-surface presentation, and because the focus of this report is methodological, we present three of these candidates here as illustrative examples. They were selected to span HLA alleles, peptide chemistries, and prior detection status, rather than as a detection-rate survey of the full set. For example, the peptide TIHDIILECV, an HLA-A∗02:01-restricted epitope which had previously been targeted for MS detection, and for which the acquired signal was too weak to confirm detection with certainty (37), was now confidently detected in CaSki cells. Initially observed at trace levels with a notable RT shift, a subsequent replicate yielded a much stronger signal, and a final confirmation experiment with a SIL internal standard resolved the minor RT discrepancy and unequivocally validated its identity (Fig. 5*B*). We also achieved, to our knowledge, the first MS-based detection of ISEYRHYCY, a novel HLA-A∗01:01-restricted peptide, in the Goerke cell line. The initial signal was extremely weak, but subsequent focused runs provided clearer evidence. A final spike-in experiment, though also yielding a very weak signal, surpassed our NSA threshold and confirmed the absolute retention time match (Fig. 5*C*). Finally, we report the MS detection of the HLA-A∗11:01-restricted peptide CVYCKQQLLR in W12-20861 cells for the first time. This target was affected by co-eluting off-target ions but was strongly detected in initial runs, albeit with an RT shift. Spike-in confirmation resolved this discrepancy and validated the detection (Fig. 5*D*). Across all three targets, the endogenous signals were at exceptionally low intensity; in particular, no precursor signal was observed in the MS1 survey scans. This is characteristic of the ultra-low-abundance regime that optiPRM was designed for: as we previously reported when introducing optiPRM, precursor ion chromatograms extracted from full MS scans did not reach the sensitivity of the corresponding fragment ion chromatograms (9).

**Fig. 6:**
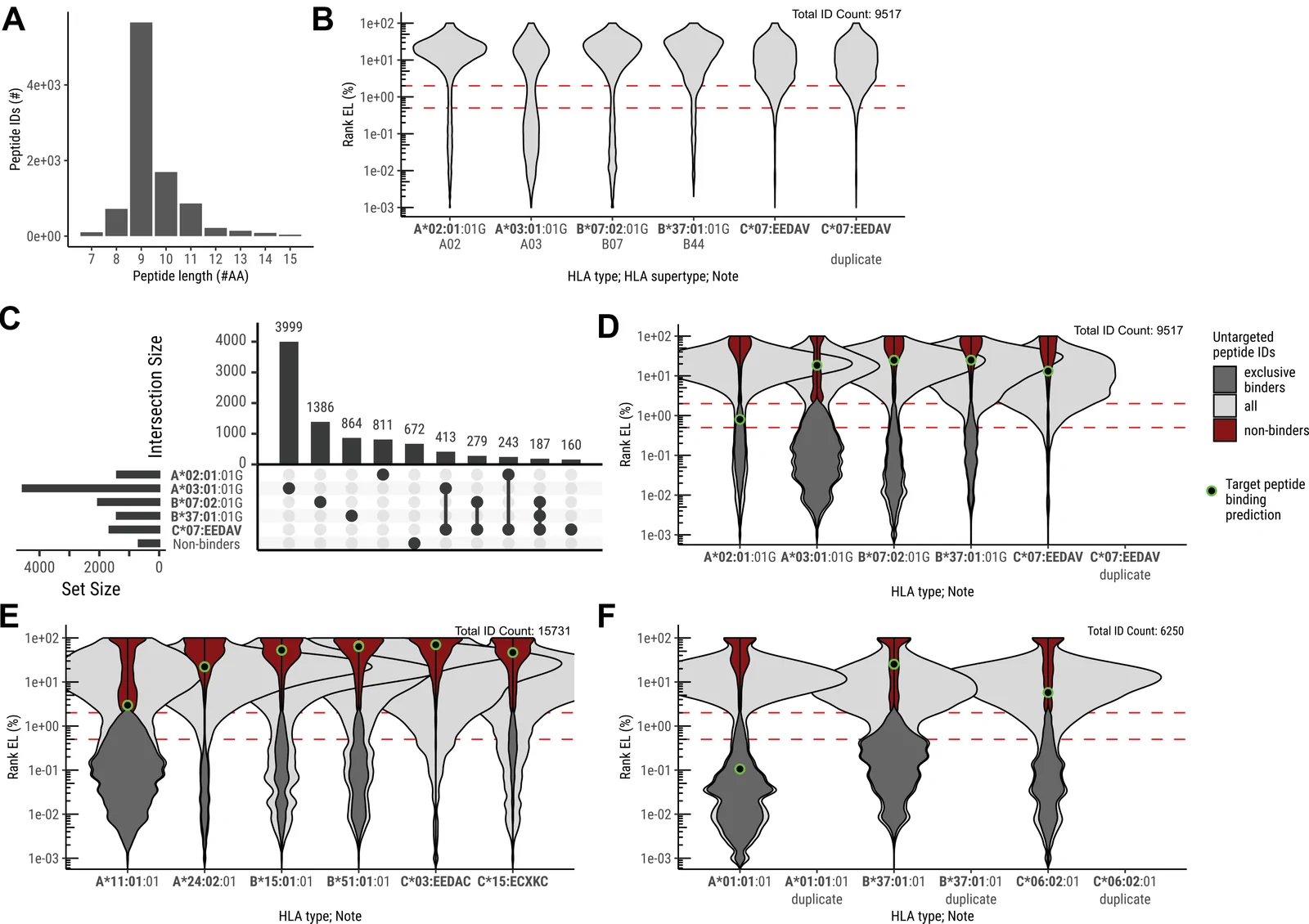
**Contextualizing targeted detection with untargeted immunopeptidomics.***A–D*, results for cell line CaSki. (*A*) Peptide length distribution of all detected peptides. *B*, the eluted ligand (EL) predictions for each peptide to each of the HLA allotypes of the cell line. Lower ranks indicate better binding prediction. The dashed red lines indicate the recommended *top* 0.5% rank threshold for strong HLA binders and the 2% rank threshold for weak HLA binders. The x-axis specifies the HLA-allele and the matching supertype, if any; the four-digit HLA allele typing is shown in bold to emphasize the most relevant information. *C*, upset plot for all predicted HLA-allele:peptide matches according to the weak binder cutoff. *D*, Enhanced violin plot integrating data from (*B*) and (*C*) with focus on predicted binder populations. Exclusive binders to a given HLA allotype (according to weak binder cutoff) and non-binders are highlighted. *Dashed red lines* indicate binding thresholds as in *B*. *Green* dots are an overlay of peptide TIHDIILECV, which was not among the untargeted detections in CaSki, but was detected via targeted MS. *E*, overlay of peptide CVYCKQQLLR (green dots) over the untargeted immunopeptidome of W12-20861 (enhanced violin plot). As in *D*, this peptide was not among the untargeted detections but was detected via targeted MS. *F*, overlay of peptide ISEYRHYCY (*green dots*) over the untargeted immunopeptidome of Goerke (*enhanced violin plot*). As in *D*, this peptide was not among the untargeted detections but was detected via targeted MS. The shared plotting legend shown at panel (*D*) applies to panels (*D*–*F*).

To contextualize these targeted detections and independently validate their predicted HLA restrictions, we performed untargeted immunopeptidomics analysis using data-independent acquisition (DIA) on the same cell lines. Critically, none of the three targeted peptides were detected by untargeted DIA across the respective cell lines (CaSki, Goerke, and W12-20861; Fig. 6, *D*–*F*), underscoring the superior sensitivity of our optimized targeted workflow. Analysis of the global immunopeptidome of the CaSki cell line revealed a typical peptide length distribution (Fig. 6*A*) and a MS-detection landscape dominated by peptides predicted to bind HLA-A∗03:01 when using a standard weak binder cutoff of 2% netMHCpan 4.1b Rank EL (Fig. 6, *B* and *C*). By overlaying the binding prediction for the detected peptide TIHDIILECV onto this landscape, we confirmed that its predicted restriction to HLA-A∗02:01 represents the most plausible hypothesis, as it falls well within the distribution of other A∗02:01 binders and is a poor binder to all other expressed alleles (Fig. 6*D*). This contextual analysis was particularly insightful for the peptide CVYCKQQLLR. Although this peptide was previously confirmed as a binder in *in vitro* experiments (21), it was not predicted as a binder by NetMHCpan. However, analysis of the untargeted data from W12-20861 cells shows that HLA-A∗11:01 is the only plausible restricting allele (Fig. 6*E*). Supporting this conclusion, the population of predicted "non-binders" for this cell line shows a distinct shoulder clustering just above the 2% Rank EL threshold specifically for A∗11:01, confirming that the *in silico* model is too stringent for this allele and that more true binders exist beyond the commonly used cutoff, consistent with previous observations (21). This observation strengthens our conclusion that CVYCKQQLLR is a *bona fide* A∗11:01-presented epitope and highlights the importance of pursuing targets beyond strict *in silico* prediction thresholds. Finally, overlaying ISEYRHYCY onto the untargeted immunopeptidome of the Goerke cell line placed it clearly within the distribution of HLA-A∗01:01 binders, independently supporting its predicted A∗01:01 restriction (Fig. 6*F*).

## Discussion

In this study, we established and applied an ultra-sensitive, optimized targeted immunopeptidomics workflow, termed optiPRM+. This enabled the confident identification and validation of three previously unconfirmed or novel HPV16 E6/E7-derived peptides presented by HLA class I molecules on various cancer cell lines. Using these very-low-abundance viral peptides as a test case, we demonstrate a practical advance for sensitivity-limited targeted immunopeptidomics.

A cornerstone of our targeted assay development was the systematic, empirical characterization of each synthetic target peptide prior to biological sample analysis. Inclusion list-driven data-dependent acquisition (iDDA) allowed for an in-depth analysis of the chromatographic behavior and fragmentation patterns of all targets, avoiding the stochastic sampling issues inherent to conventional DDA. This was complemented by direct infusion-mass spectrometry (DI-MS) for per-peptide collision energy optimization. This rigorous upfront characterization is invaluable for building robust and sensitive PRM assays, ensuring that no critical features are overlooked, which is a common risk in standard DDA-based discovery workflows.

While the acquisition parameters and their individual effects are well established (31, 32, 33), they are conventionally held near balanced defaults to preserve cycle time across multiplexed targets. Our contribution is to show how far they can be pushed when few peptides are targeted: resolution up to 480,000, injection times to 1000 ms, and the narrowest recommended isolation window together improved signal-to-noise and spectral matching for trace analytes, with AGC the sole parameter exhibiting a clear ceiling consistent with overfilling. These "slow-scanning" settings define a peak-sensitivity regime for small target panels rather than a new parameter space. While we used a HeLa digest as a complex matrix for these optimization experiments, which is less complex than a typical immunopeptidomics eluate, we posit that this more challenging application also benefits from these settings, where maximizing specificity and ion collection time is paramount for distinguishing signal from noise.

We further demonstrated the profound benefit of per-peptide collision energy (CE) optimization, a central component of the optiPRM workflow (9). This is particularly critical for the non-tryptic peptides characteristic of the immunopeptidome, which often lack the C-terminal basic residue that typically ensures predictable fragmentation. We observed that precursors carrying a net charge (z) greater than their total number of basic amino acids (R, K, H) frequently required unusually low optimal CEs. We speculate that this phenomenon arises because, in the absence of sufficient basic sites to sequester protons, the charge is distributed more diffusely across the peptide backbone or onto less basic side chains. This may destabilize the peptide, making it susceptible to fragmentation at lower energies, or alter fragmentation pathways away from the canonical b- and y-ions. By tailoring the CE to each precursor, we significantly improved spectral quality and sensitivity for these challenging targets.

The collision-energy advantage central to optiPRM is, by its nature, available only to targeted acquisition. PRM delivers a collision energy tuned to each precursor based on empirical optimization; DDA can apply a formulaic energy derived from the known precursor mass and charge; and DIA, in which an isolation window does not correspond to a single precursor, must assume a charge state representative of that window and typically applies stepped collision energies within a conservative consensus range that is broadly effective but not target-specific. Consequently, the per-peptide collision energies optimized here (Fig. 1), particularly the unusually low energies required by precursors whose charge exceeds their basic-residue count, can be exploited in full only by the targeted component of the workflow.

To manage the inherent chromatographic variability in complex biological runs, we implemented an RT alignment strategy based on a standard set of spiked-in peptides. This approach allowed us to confidently match target detections to their assay-external SIL references without resorting to spiking the SIL peptides into every sample, thereby avoiding potential issues with light-isotope contamination (35, 36) and conserving instrument time. Conceptually, the XIC alignment for visual comparison to the reference is very close to the commonly used iRT normalization that is well accepted for peptide identification (38). Our approach reflects our requirement to diagnose XIC alignment while retaining the original RT axis. While this alignment strategy has its limitations, and direct spike-in of a titrated reference remains the gold standard for absolute RT confirmation, our approach proved highly effective for initial discovery and validation. To achieve absolute certainty, we performed spike-in validation for all detected peptides. This final validation step confirmed our initial detections in all cases, underscoring the robustness of our overall workflow. The rigorous use of negative controls, such as monitoring for LC carryover and SIL peptide contamination, was essential for ensuring the validity of our low-level detections. Ultimately, our study highlights the powerful synergy between targeted and untargeted approaches, as combined here in the optiPRM+ workflow: the original targeted optiPRM component provided the sensitivity required for the initial discovery and validation of HPV16-derived viral epitopes, while the untargeted DIA analysis provided a global view of the immunopeptidome for each cell line, which was crucial for contextualizing our targeted identifications and validating their HLA restrictions.

The MS-based verification of three HPV16-derived epitopes, two of which, to our knowledge, were detected by MS for the first time and one of which (TIHDIILECV) was only weakly detected by MS before (37), provides the field with high-confidence targets for the development of epitope-centric immunotherapies. Unlike targets derived purely from prediction algorithms or indirect immunological assays, these peptides are confirmed to be naturally processed and presented on the surface of cancer cells, making them biologically relevant candidates for therapeutic vaccines or TCR-based therapies. Our confirmation of TIHDIILECV, a peptide for which previous MS evidence was inconclusive (37) and which explicitly escaped detection in recent state-of-the-art untargeted immunopeptidomic profiling (19), solidifies its status as a *bona fide* public epitope (39). In contrast to a previous study from our lab which detected a different set of peptides using an older MS3-based technique (40), our current work successfully identified different epitopes, highlighting how orthogonal methodologies can uncover distinct portions of the immunopeptidome. The discrepancy underscores the complexity of immunopeptidomics and the significant impact of evolving sample preparation and mass spectrometry technologies on the detectable peptide repertoire. Despite the gains in sensitivity, the absolute detection limit of our workflow in terms of peptide copies per cell is peptide-dependent and remains unknown. Consequently, it is likely that epitopes that are of very low abundance or possess unfavorable physicochemical properties for MS detection are still being missed. The threshold of antigen density required for T cell recognition and subsequent tumor cell lysis is an area of active research, and it cannot be inferred whether epitopes escaping MS detection for either reason would be therapeutically actionable. This can only be answered by comprehensive immunogenicity testing of the novel mass spectrometry-validated epitopes.

As a targeted approach, optiPRM+ is subject to the same fundamental constraint as any PRM method: the required cycle time, and hence the degree of multiplexing, places a ceiling on the number of peptides that can be monitored at peak sensitivity, with the practical impact depending on how many targets co-elute. In our hands, panels of up to roughly one hundred peptides were routinely feasible in a first round, and a complete assay, from synthetic-peptide characterization through acquisition to analysis, can be configured within about 1 week when synthetic peptides are in hand. Relative to discovery DDA and DIA, the targeted-first design spends instrument time on MS2 of defined targets rather than on repeated MS1 precursor sampling; even sensitive DDA methods using long injection times must still detect the precursor within a complex MS1 survey scan, whereas optiPRM+ does not depend on MS1 precursor detection, which is the basis of its sensitivity advantage for the ultra-low-abundance peptides studied here. Within the landscape of targeted immunopeptidomics, optiPRM+ occupies a middle ground: approaches such as SureQuant achieve the highest quantitative rigor by calibrating a stable isotope-labeled standard for every target and cycling the heavy standard in parallel (41), whereas optiPRM+ prioritizes detection sensitivity and characterizes each target against its synthetic SIL variant in a scalable fashion that does not require per-run spike-in; owing to the assay-external SIL reference, detections remain high-confidence, and an optional focused SIL spike-in can readily be added when absolute confirmation is required.

We further note that all three illustrative epitopes are cysteine-containing, which reflects the cysteine-rich nature of the HPV16 oncoproteins E6 and E7 (42) rather than a detection bias of the workflow. The high reactivity of cysteine residues during sample preparation is known to hamper the MS detection of specific viral targets such as TIHDIILECV (19). Therefore, standard reduction and alkylation steps are crucial to stabilize these peptides, whereas the targeted assays must support confident detection of these cysteine-containing targets among many candidates.

In conclusion, the optiPRM+ workflow provides a practical, purpose-built approach for the sensitive and specific detection of low abundant HLA-presented peptides. By applying this highly optimized strategy to viral antigens of known low abundance, we added MS-validated HPV16 ligands not previously confirmed by MS (19, 37, 40), providing validated, therapeutically relevant epitopes for the rational design of next-generation cancer immunotherapies. This work underscores the power of purpose-built, optimized targeted mass spectrometry to bridge the gap between genomic prediction and the direct observation of the tumor antigen landscape.

## Declaration of Generative AI and AI-Assisted Technologies in the Manuscript Preparation Process

During the preparation of this work, the authors used Google Gemini for assistance with language editing and stylistic refinement and to support the creation of schematic illustrations. After using this tool/service, the authors reviewed and edited the content as needed and take full responsibility for the content of the published article.

## Data Availability

The untargeted mass spectrometry data have been deposited to the ProteomeXchange Consortium via the PRIDE (43) partner repository with the dataset identifier PXD074199.

Reviewer Access: Username: reviewer_pxd074199@ebi.ac.uk Password: 2qNcNx8qT7jk.

The chromatograms and rawfiles of the targeted assays are available via PanoramaWeb Public (44) under the permanent link https://panoramaweb.org/H6Nuxj.url.

Reviewer Access: Email: panorama+reviewer403@proteinms.net Password: w38z#qDHL!∗p0l.

## Supplemental data

This article contains supplemental data.

## Conflict of interest

The authors declare that they have no conflicts of interest with the contents of this article.
