# Supplementary material for: Systematic Workflow Optimization for Ultra-sensitive Targeted Immunopeptidomics
Source: Mol Cell Proteomics. 2026 Aug 4;25(9):101634. doi: 10.1016/j.mcpro.2026.101634 (PMC13545375; doi:10.1016/j.mcpro.2026.101634)
Supplement: Supplementary file 1 [file mmc1.pdf]

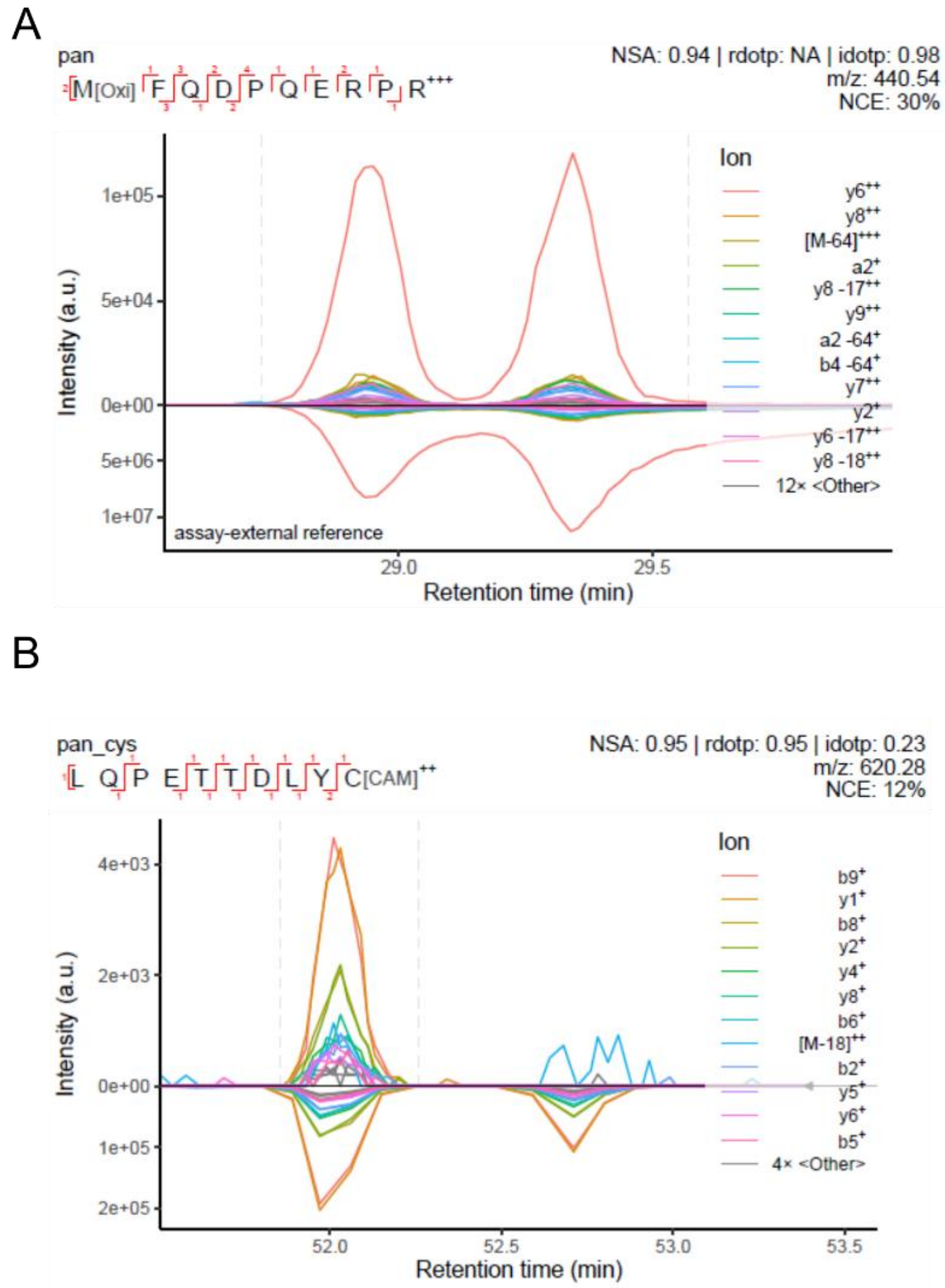

Figure S1. Peptides showing multiple peaks. (A) Methionine-containing peptide AYVHM[Ox]VTHF. (B) Proline-containing peptide LQPETTDLYC showing multiple peaks only in the synthetic reference.

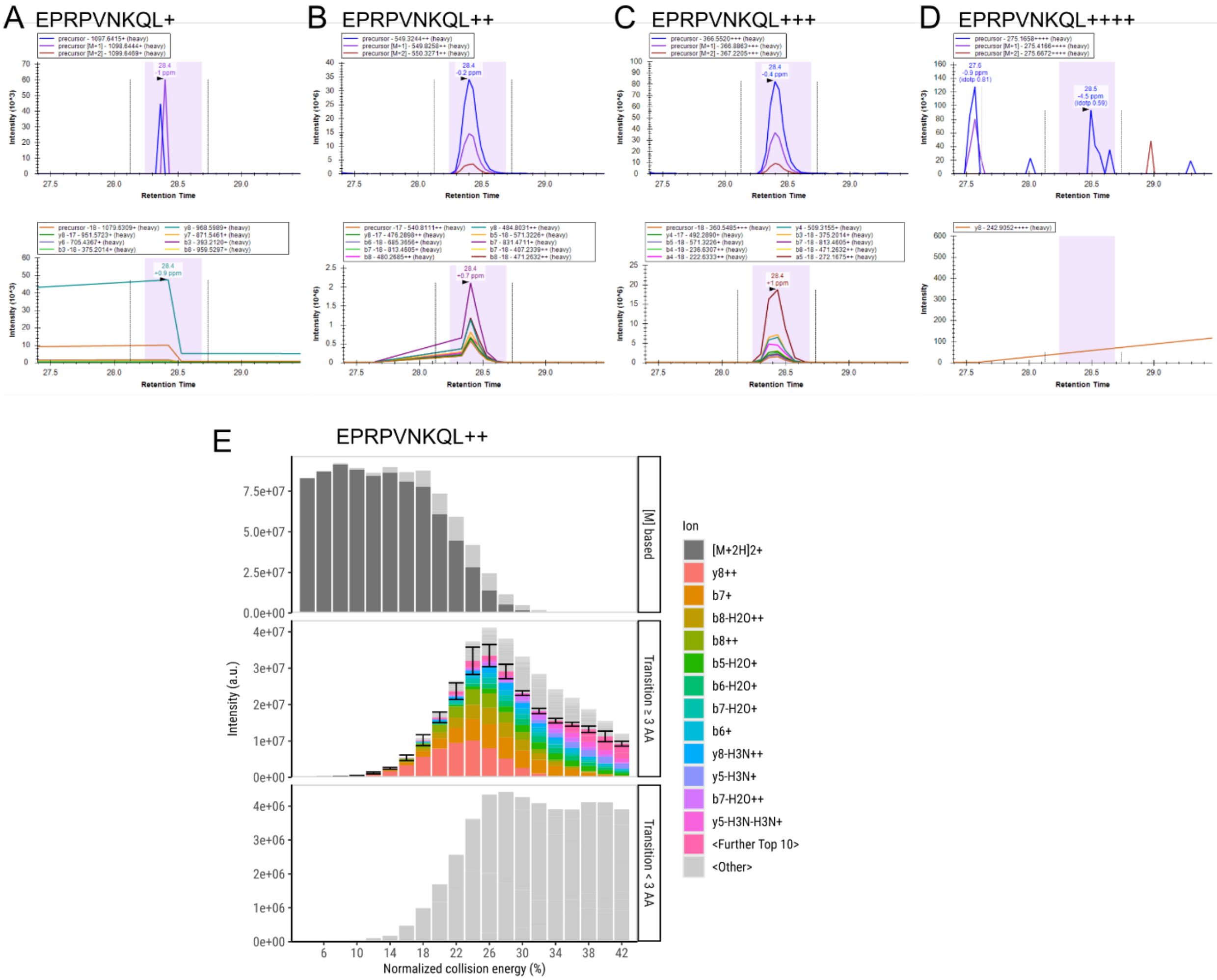

Figure S2. LC-MS precursor intensities of peptide EPRPVNKQL. (A-D) Precursor charge states +1 to +4 that were targeted via inclusion list DDA. (E) Stacked quantification of MS2 signal acquired via DI-MS for peptide EPR[+10]PVNKQL[+7] at precursor charge state +2 over a range of normalized collision energies (NCEs). For each NCE, five spectra were acquired and mean ion intensities are shown. The main focus is on highly specific fragments of length ≥ 3 AA and the most dominant fragments are colored. Ions of lower specificity are separated as precursor ([M]-based) and of length < 3 AA. Confidence intervals are the standard deviation of the sum of the top 10 ions.

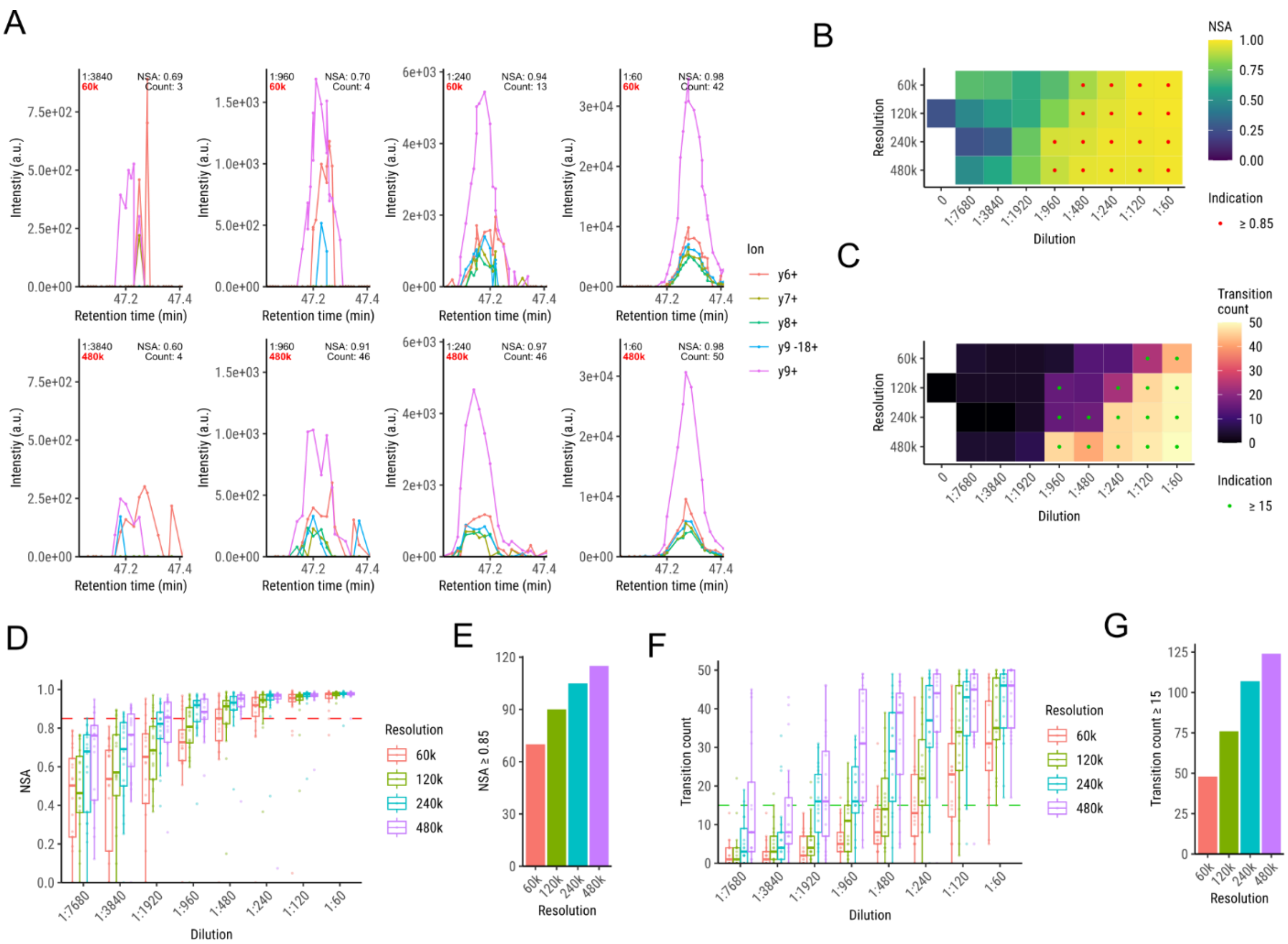

Figure S3. Test series on the effect of MS resolution on detection sensitivity. The PROCAL synthetic peptide kit is diluted against a consistent complex matrix of tryptic HeLa digest. (A) Exemplary XIC for the precursor TFGTETFDTFK++. Annotated at the top left of each panel is the dilution and MS resolution used. Annotated at the top right are the NSA and total count of detected transitions. (B) Full panel of NSA results for the precursor in (A). (C) Full panel of transition counts for the precursor in (A). (D) The distribution of NSA that were measured for the +2 precursor of each peptide at all dilutions and resolutions. The dashed red line indicated the threshold of NSA ≥ 0.85. (E) Sum of all events in (D) that passed the threshold. (F) The transition counts that were measured for all precursors at all dilutions and resolutions. The dashed red line indicates the threshold of ≥ 15. (G) Sum of all events in (F) that passed the threshold.

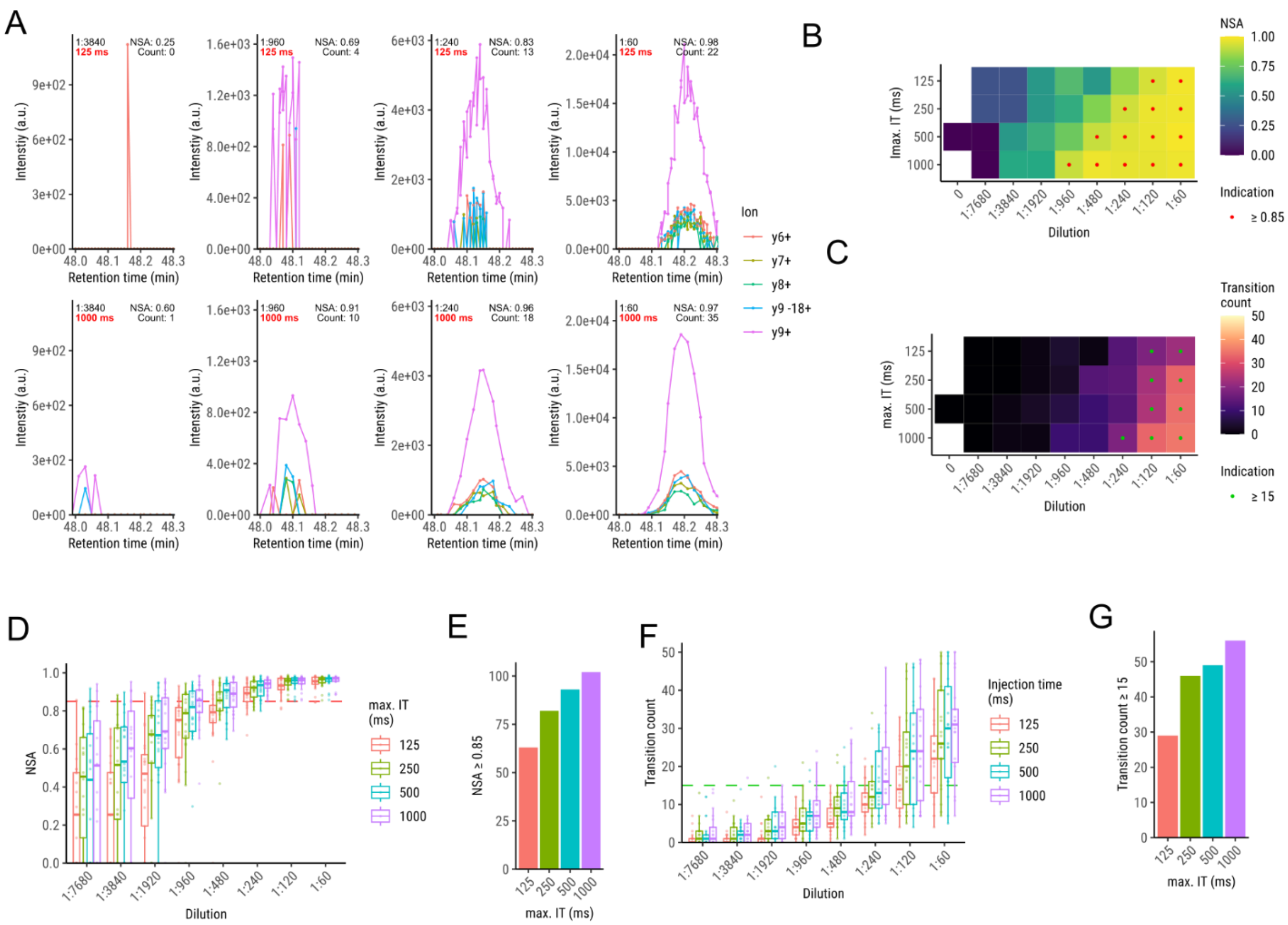

Figure S4. Test series on the effect of maximum MS2 injection time on detection sensitivity. The synthetic PROCAL peptide kit is diluted against a consistent complex matrix of tryptic HeLa digest. (A) Exemplary XIC for the precursor TFGTETFDTFK++. Annotated at the top left of each panel is the dilution and max. IT used. Annotated at the top right are the NSA and total count of detected transitions. (B) Full panel of NSA results for the precursor in (A). (C) Full panel of transition counts for the precursor in (A). (D) The distribution of NSA that were measured for the +2 precursor of each peptide at all dilutions and max. ITs. The dashed red line indicated the threshold of NSA ≥ 0.85. (E) Summary of all events in (D) that passed the threshold. (F) The transition counts that were measured for all precursors at all dilutions and max. ITs. The dashed red line indicates the threshold of ≥ 15. (G) Summary of all events in (F) that passed the threshold.

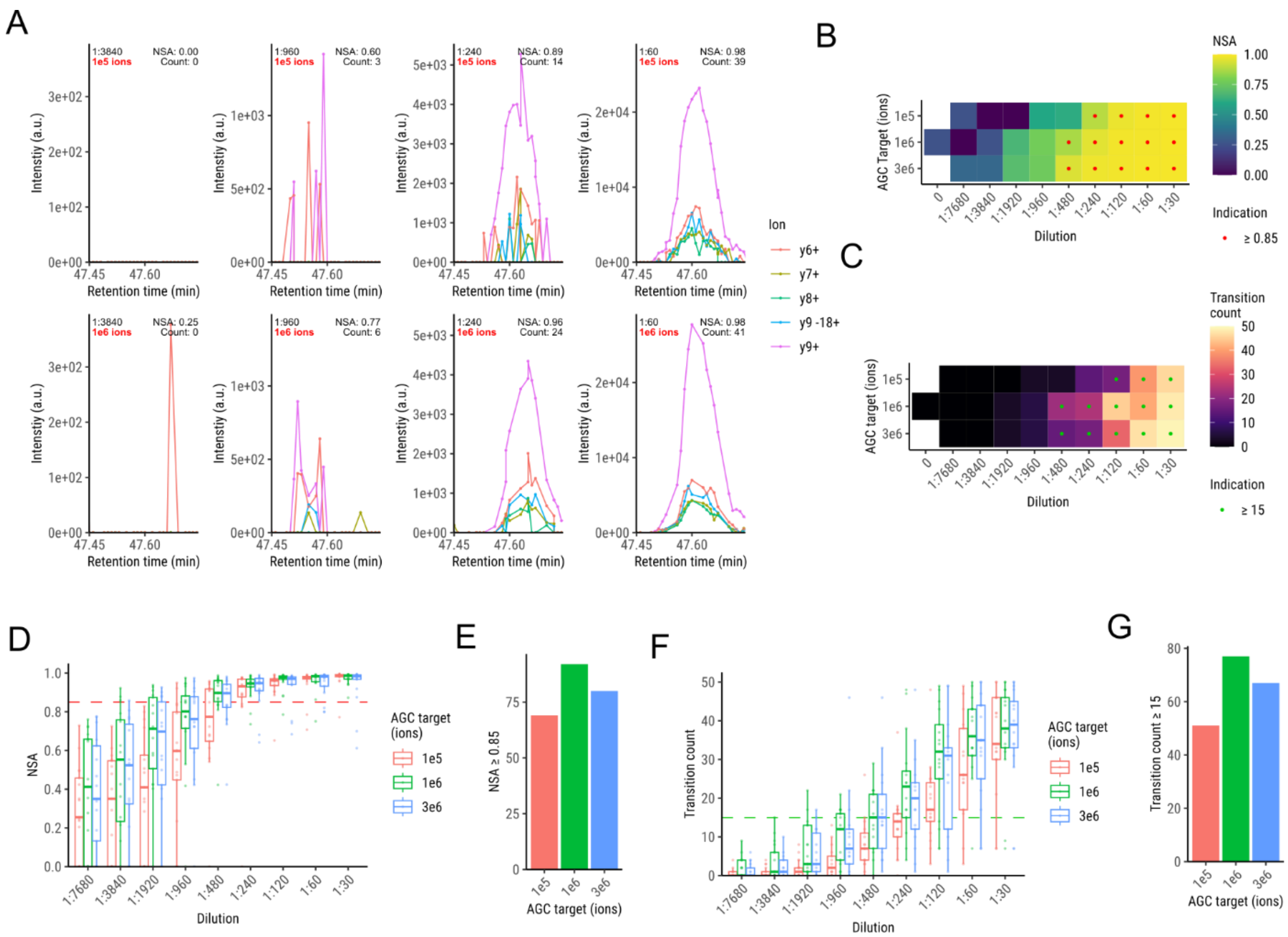

Figure S5. Test series on the effect of AGC target on detection sensitivity. The synthetic PROCAL peptide kit is diluted against a consistent complex matrix of tryptic HeLa digest. (A) Exemplary XIC for the precursor TFGTETFDTFK++. Annotated at the top left of each panel is the dilution and MS resolution used. Annotated at the top right are the NSA and total count of detected transitions. (B) Full panel of NSA results for the precursor in (A). (C) Full panel of transition counts for the precursor in (A). (D) The distribution of NSA that were measured for the +2 precursor of each peptide at all dilutions and resolutions. The dashed red line indicated the threshold of NSA ≥ 0.85. (E) Summary of all events in (D) that passed the threshold. (F) The transition counts that were measured for all precursors at all dilutions and resolutions. The dashed red line indicates the threshold of ≥ 15. (G) Summary of all events in (F) that passed the threshold.

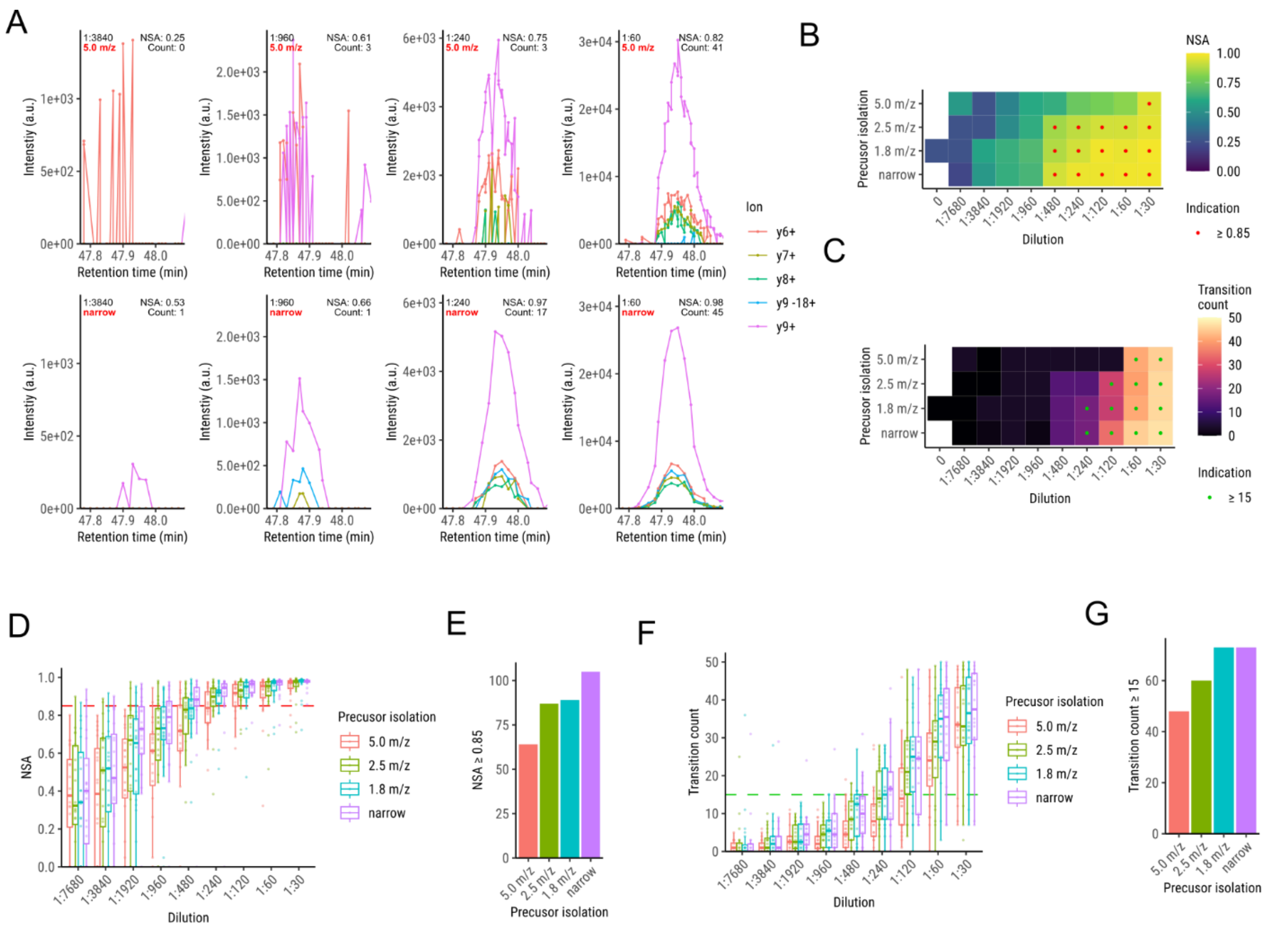

Figure S6. Test series on the effect of precursor isolation window on detection sensitivity. The synthetic PROCAL peptide kit is diluted against a consistent complex matrix of tryptic HeLa digest. (A) Exemplary XIC for the precursor TFGTETFDTFK++. Annotated at the top left of each panel is the dilution and isolation window used. Annotated at the top right are the NSA and total count of detected transitions. (B) Full panel of NSA results for the precursor in (A). (C) Full panel of transition counts for the precursor in (A). (D) The distribution of NSA that were measured for the +2 precursor of each peptide at all dilutions and isolation windows. The dashed red line indicated the threshold of NSA ≥ 0.85. (E) Summary of all events in (D) that passed the threshold. (F) The transition counts that were measured for all precursors at all dilutions and isolation windows. The dashed red line indicates the threshold of ≥ 15. (G) Summary of all events in (F) that passed the threshold.

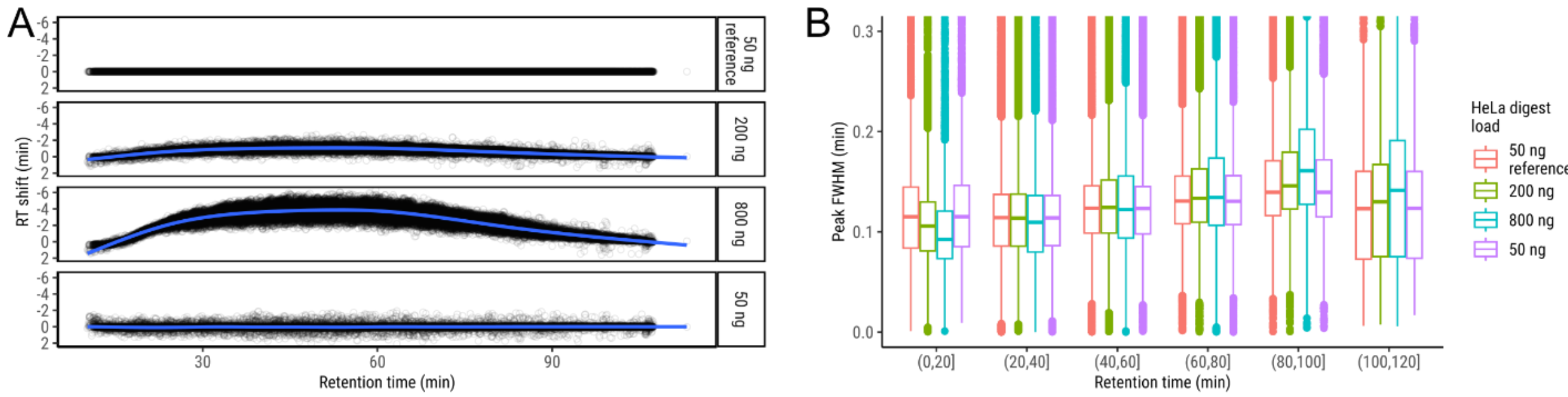

Figure S7. Systematic RT shifts as a function of peptide load on the LC system. (A) RT shift observed for peptides in untargeted acquisition of different amounts of tryptic HeLa digest. Only peptides detected in all runs are shown. The initial run at the top serves as the reference for the RT shift. Subsequent LC-MS were performed as shown from top to bottom. The method for the blue trendline is a generalized additive model (GAM) in R with formula y ~ s(x, bs = 'cs'). (B) Peak full width at half maximum (FWHM) observed in the runs in (A).

Table S1. Targeted PRM precursor and transition list. Precursor ions and their monitored fragment (transition) ions for each analyte characterized or detected in this study, comprising the collision-energy/parameter-optimization peptides, the synthetic illustrative peptides shown in the figures, and the validated HPV16 epitopes. Columns: precursor (peptide), precursor m/z, transition (fragment ion), transition m/z.

Table S2. DIA isolation-window scheme. The 44 variable precursor isolation windows used for the untargeted DIA method, with center m/z, precursor charge, isolation-window width, and HCD collision energy. The method cycle time was set to Automatic (dynamic), with a 3e6 AGC target.

Table S3. Fragment-ion intensities underlying the NSA calculation. Per-fragment extracted-ion-chromatogram areas, measured intensities, and reference-library intensities used to compute the normalized spectral contrast angle (NSA) for the synthetic peptide TFGTETFDTFK (z=+2) shown in Figure 3B–C, across dilutions and MS2 resolutions.
